# Draft Genome Sequence of a Clinical Isolate of *Serratia marcescens*, Strain AH0650_Sm1

**DOI:** 10.1128/genomeA.01007-15

**Published:** 2015-09-03

**Authors:** Yu Wan, Claire L. Gorrie, Adam Jenney, Mirjana Mirceta, Kathryn E. Holt

**Affiliations:** a Department of Biochemistry and Molecular Biology, Bio21 Molecular Science and Biotechnology Institute, University of Melbourne, Victoria, Australia; b Centre for Systems Genomics, University of Melbourne, Victoria, Australia; c Department of Microbiology and Immunology, Peter Doherty Institute for Infection and Immunity, University of Melbourne, Victoria, Australia; d Department of Infectious Diseases and Microbiology Unit, The Alfred Hospital, Victoria, Australia

## Abstract

*Serratia marcescens* strain AH0650_Sm1 is a clinical multidrug-resistant isolate from Australia. Here, we report its annotated draft genome comprising 20 contigs. We identified chromosomal antimicrobial resistance genes including a *tet*(41) variant, an *aac(6′)-Ic* variant, *ampC*, a metallo-beta-lactamase, and several putative multidrug efflux pumps, as well as a novel prophage.

## GENOME ANNOUNCEMENT

The Gram-negative, facultative anaerobic and motile bacillus *Serratia marcescens* is an opportunistic human pathogen and a member of the *Enterobacteriaceae* (1, 2). It is ubiquitous in nature and has been recognized since the 1950s as an etiological agent of nosocomial infections (3), including bacteremia, pneumonia, meningitis, myocarditis, endocarditis, respiratory tract infections, urinary tract infections, and wound infections (4–6). Moreover, *S. marcescens* can pose a serious obstacle to antimicrobial treatment of infections due to its intrinsic and acquired resistance to a wide range of antimicrobials (7, 8).

*S. marcescens* strain AH0650_Sm1 was isolated from the sputum of a pneumonia patient at the Alfred Hospital Intensive Care Unit in Melbourne, Australia, on 20 March 2014. Antimicrobial susceptibility tests via the Vitek 2 system (bioMérieux, France) showed that AH0650_Sm1 was resistant to ampicillin (MIC 16), amoxicillin-clavulanic acid (MIC ≥32), ticarcillin-clavulanic acid (MIC ≤8), piperacillin-tazobactam (MIC ≤4), tobramycin (MIC 4), nitrofurantoin (MIC 256), and cefazolin (MIC ≥64); intermediately resistant to cefoxitin (MIC 16); and susceptible to amikacin (MIC ≤2), ceftriaxone (MIC ≤1), ceftazidime (MIC ≤1), cefepime (MIC ≤1), meropenem (MIC ≤0.25), gentamicin (MIC ≤1), ciprofloxacin (MIC ≤0.25), norfloxacin (MIC ≤0.5), trimethoprim (MIC ≤0.5), and trimethoprim-sulfamethoxazole (MIC ≤20). The unit of all MICs is µg/ml.

Whole-genomic DNA was extracted using phenol-chloroform and the Phase Lock Gel protocol (5PRIME), with some minor adaptations, and a barcoded library was prepared using the Nextera XT kit (Illumina, USA). Paired-end sequencing was performed at the Australian Genome Research Facility with the Illumina HiSeq 2500 system, generating 1,858,192 read pairs (2 × 125 bp) yielding 90× coverage. Reads were filtered for an average Phred quality ≥30 and assembled *de novo* using SPAdes version 3.5.0 (9) with *k*-mer lengths of 21, 33, 55, 77, and 99. SSPACE version 3.0 (10) was used for scaffolding, and GapFiller version 1.10 (11) was used for filling gaps. Contigs were further extended and reordered using AlignGraph version 27062014 (12) and ABACAS version 1.3.1 (13), respectively, with reference to the finished chromosome sequence of *S. marcescens* Db11 (RefSeq accession no. NZ_HG326223). Contigs shorter than 200 bp were removed. The final assembly was annotated using Prokka version 1.11 (14). Antimicrobial resistance genes and plasmid replicons were screened using SRST2 (15) with databases from ARG-ANNOT (16) and PlasmidFinder (17). PHAST (18) was used for identifying prophage regions. These genomic features were further investigated using nucleotide and protein BLAST.

This draft genome contains 5,201,657 bp assembled into 20 contigs with an *N*_50_ of 946 kbp. The genomic annotation includes 4,734 protein coding sequences, 88 tRNA genes, 22 rRNA genes, and one tmRNA gene. Genes related to antimicrobial resistance were identified, including variants of *tet*(41) and *tetR*(41) (8), an *aac(6′)-Ic* (19) variant, *ampC*, *ampR*, and those encoding a metallo-beta-lactamase and several multidrug efflux pumps. A putative novel prophage (31.8 kbp) was identified, which shared 75% identity with *Salmonella* phage FSL SP-004 (RefSeq GenBank accession no. NC_021774) along 60% of its length. No plasmid replicon was detected.

### Nucleotide sequence accession numbers. 

This whole-genome shotgun project has been deposited at DDBJ/EMBL/GenBank under the accession number LFJS00000000. The version described in this paper is the first version, LFJS00000000.1.
